# Associations between cerebrospinal fluid biomarkers related to endothelial dysfunction and Alzheimer’s disease

**DOI:** 10.3389/fnagi.2026.1744647

**Published:** 2026-09-21

**Authors:** Yang-Ke Zhu, Qiu-Jie Zhu, Lan-Yang Wang, He-Ying Hu, Xue-Jie Wang, Zi-Hao Zhang, Wei Zhang, Lan Tan, Hao Hu

**Affiliations:** 1 Department of Neurology, Qingdao Municipal Hospital, Qingdao University, Qingdao, China; 2 Department of Psychosomatics and Psychiatry, Zhongda Hospital, School of Medicine, Southeast University, Nanjing, Jiangsu, China; 3 Department of Neurology, Qingdao Hospital, University of Health and Rehabilitation Sciences (Qingdao Municipal Hospital), Qingdao, China; 4 Institute of Neurology, Jiangxi Academy of Clinical Medical Sciences, The First Affiliated Hospital, Jiangxi Medical College, Nanchang University, Nanchang, Jiangxi, China

**Keywords:** Alzheimer’s disease, cognition, endothelial dysfunction, MMP-10, tau biomarkers

## Abstract

**Background:**

Although associations between overt vascular injury and Alzheimer’s disease (AD) have been reported, the role of subclinical vascular injury in dementia pathogenesis remains unclear. Cerebrospinal fluid (CSF) biomarkers related to endothelial dysfunction may capture subclinical vascular impairment and provide insights into early AD pathogenesis.

**Methods:**

We examined associations of ten CSF biomarkers (PDGFRβ, Osteopontin, VCAM-1, E-selectin, MMP-2, MMP-10, MMP-3, MMP-12, MMP-9/TIMP-1 ratio and Periostin) with AD core biomarkers, brain volume, cognitive function, and risk of incident AD among 444 participants. Multivariable linear regression, linear mixed-effects models, Cox proportional hazards models, mediation analyses, and 3-year time-dependent ROC analyses were applied. All models were adjusted for demographic factors, *APOE* ε4 status, and vascular risk factors.

**Results:**

Seven of ten biomarkers were significantly associated with CSF tau biomarkers, whereas two biomarkers were associated with CSF Aβ_42_. Mediation analyses suggested that CSF tau biomarkers statistically accounted for a greater proportion of the associations between MMP-10 and cognition than did Aβ_42_. Among the biomarkers examined, MMP-10 showed the most consistent associations across AD-related outcomes, including lower CSF Aβ_42_, higher CSF tau biomarkers, poorer cognitive performance, greater longitudinal brain atrophy, and increased risk of incident AD dementia.

**Conclusion:**

Cerebrospinal fluid biomarkers related to endothelial dysfunction and vascular injury were more consistently associated with CSF tau biomarkers than with CSF Aβ_42_ in non-demented individuals. MMP-10 showed the most consistent pathological, cognitive, neuroimaging, and prognostic associations and provided incremental predictive information for incident AD dementia. These findings support further investigation of endothelial dysfunction-related and vascular remodeling pathways as potential correlates of AD progression.

## 1 Introduction

Alzheimer’s disease (AD) is recognized as the leading neurodegenerative condition contributing to dementia and poor cognition in older adults. The characteristic pathological features of AD involve excessive deposition of amyloid-β (Aβ) in extracellular plaques and the accumulation of tau protein as intracellular neurofibrillary tangles (Blennow et al., 2006; Zlokovic et al., 2010; De Kort et al., 2022). However, accumulating evidence suggests that AD pathogenesis involves multiple interacting mechanisms beyond these classical biomarkers, among which vascular dysfunction has emerged as an important contributor to disease development and progression. Endothelial dysfunction, considered an early manifestation of vascular abnormalities, disrupts vascular homeostasis and has been implicated in AD-related pathological changes and cognitive decline (Andjelkovic and Pachter, 1998; Buée et al., 1994; Custodia et al., 2021, 2023; Grammas, 2011; Toledo et al., 2013; Zlokovic et al., 2010; Zlokovic, 2005). As key structural and functional components of the blood-brain barrier (BBB), endothelial cells regulate cerebral blood flow and maintain BBB integrity (Garcia-Gallardo and Campbell, 2025; Yi et al., 2025). Although endothelial dysfunction is increasingly recognized as relevant to AD, there is currently no universally accepted CSF biomarker panel that exclusively defines endothelial dysfunction in this context. Moreover, endothelial dysfunction is closely intertwined with multiple neurovascular processes, including BBB impairment, extracellular matrix remodeling, vascular inflammation, pericyte injury, and dysfunction of the broader neurovascular unit. Therefore, to capture different aspects of endothelial dysfunction-related and vascular injury-related pathological processes, we adopted a functional and relatively broad definition of CSF biomarkers related to endothelial dysfunction. In the present study, the term “biomarkers related to endothelial dysfunction” refers to biomarkers that have been functionally linked to endothelial activation, vascular dysfunction, BBB impairment, extracellular matrix remodeling, vascular inflammation, and associated cerebrovascular processes rather than biomarkers with exclusive endothelial origin. Several cerebrospinal fluid (CSF) biomarkers have been proposed as indicators of endothelial dysfunction and related vascular abnormalities in AD. Specifically, platelet-derived growth factor β (PDGFRβ), a marker of pericyte injury, is essential for maintaining cerebral microcirculation and BBB integrity (Winkler et al., 2010). Vascular cell adhesion molecule-1 (VCAM-1) and E-selectin are endothelial adhesion molecules involved in endothelial activation and leukocyte recruitment following vascular injury (Ewers et al., 2010; Kawamura et al., 1998; Neal et al., 2025; Salian et al., 2024). Osteopontin is a multifunctional glycoprotein implicated in vascular inflammation, immune responses, and tissue repair (Chellaiah and Hruska, 2003; Cho et al., 2009). Matrix metalloproteinases (MMPs), including MMP-2, MMP-3, MMP-9, MMP-10 and MMP-12, together with the MMP-9/TIMP-1 ratio, reflect extracellular matrix remodeling and BBB related processes and have been implicated in AD related vascular abnormalities (Cabral-Pacheco et al., 2020; Park et al., 2009; Yong et al., 2001). Periostin (POSTN) is an extracellular matrix-associated matricellular protein implicated in vascular injury, extracellular matrix remodeling, and inflammation (Hristovska et al., 2026). Together, PDGFRβ, Osteopontin, VCAM-1, E-selectin, POSTN, MMP-2, MMP-3, MMP-9, MMP-10, MMP-12, and the MMP-9/TIMP-1 ratio have been suggested as candidate biomarkers reflecting endothelial dysfunction-related and vascular injury-related processes (Custodia et al., 2023; Drake et al., 2021; Li et al., 2015; Vrillon et al., 2025; Wang et al., 2014).

In the 2024 NIA-AA biological framework, vascular injury has been incorporated into the etiological spectrum of AD (Høilund-Carlsen et al., 2024; Jack et al., 2024). However, currently recommended vascular biomarkers are primarily imaging-based measures, such as white matter hyperintensities detected by CT or MRI, whereas reliable fluid biomarkers remain lacking (Jack et al., 2024). Because neuroimaging abnormalities often indicate relatively advanced vascular injury, CSF biomarkers may provide a unique opportunity to detect subtle vascular alterations during the early stages of AD. Nevertheless, previous studies have mainly focused on clinically diagnosed AD patients or individual endothelial dysfunction-related biomarkers. Consequently, the relative importance of different endothelial dysfunction-related pathways and their associations with AD pathology, cognition, brain structure, and disease progression remain incompletely understood, particularly during the non-demented stages of AD. By leveraging the well-characterized ADNI cohort, this integrated analysis may help prioritize candidate fluid biomarkers of vascular dysfunction and clarify how endothelial dysfunction-related pathways are linked to AD pathology, neurodegeneration, cognitive impairment, and clinical progression before dementia onset.

Therefore, we systematically evaluated CSF biomarkers related to endothelial dysfunction in non-demented individuals. Specifically, we aimed to: (1) investigate the differences in CSF biomarkers related to endothelial dysfunction across different ATN groups; (2) examine associations between these biomarkers and AD core biomarkers, cognitive performance and brain volumes; (3) test whether AD core biomarkers partially explain the associations between endothelial dysfunction and cognition; and (4) investigate their associations with incident AD dementia risk.

## 2 Materials and methods

### 2.1 Study participants

The data utilized were sourced from the Alzheimer’s Disease Neuroimaging Initiative (ADNI) database, including participants from the ADNI-1, ADNI-GO, and ADNI-2 phases. ADNI is a large-scale, multi-center, longitudinal project aimed at improving the prediction of AD onset through the integration of clinical assessments, neuroimaging data, genetic profiles, and biochemical markers. Participants were recruited across academic centers and affiliated clinics in North America, and written informed consent was obtained from all participants.

Analyses were restricted to non-demented participants with available CSF AD biomarkers, CSF biomarkers related to endothelial dysfunction, and complete baseline covariate data. After excluding participants with missing data and those with extreme endothelial dysfunction-related biomarker values exceeding ±4 SD from the sample mean, the final cross-sectional analytic sample comprised 444 participants. Longitudinal analyses were conducted among participants with available baseline exposure and covariate data and at least one repeated follow-up assessment for the corresponding outcome, resulting in a longitudinal analytic sample of 295 participants (Supplementary Table 1). Participants were classified as cognitive normal (CN) [CDR = 0, Mini-Mental State Examination (MMSE) > 24] and mild cognitive impairment (MCI) (CDR = 0.5, MMSE > 24, objective memory loss on the education adjusted Wechsler Memory) according to the established criteria (Aisen et al., 2010). The mean follow-up duration in the longitudinal sample was 2.59 ± 1.60 years.

### 2.2 CSF biomarkers related to endothelial dysfunction and AD core biomarkers measurements

In this study, the levels of CSF PDGFRβ, Osteopontin, VCAM-1, E-selectin, MMP-2, MMP-10, MMP-3, MMP-9, MMP-12, POSTN and TIMP-1 were from the ADNI dataset “CruchagaLab CSF SOMAscan7k Protein matrix postQC” online. The CSF biomarkers related to endothelial dysfunction were quantified using the SOMAscan assay, with detailed protocols available on the ADNI website^1^. The SOMAscan platform is a high-throughput aptamer-based proteomic assay that utilizes Slow Off-rate Modified Aptamers (SOMAmers), which are chemically modified single-stranded DNA molecules designed to bind target proteins with high affinity and specificity. Following protein binding, the SOMAmer-protein complexes are isolated, and the bound aptamers are quantified using microarray-based detection, providing relative measures of protein abundance. This technology enables the simultaneous measurement of thousands of proteins across a broad dynamic range and has been extensively applied in biomarker discovery studies, including large-scale analyses within the ADNI cohort.

The levels of CSF AD core biomarkers, including amyloid-β1–42 (Aβ_42_), phosphorylated tau (P-tau), and total tau (T-tau), were measured at the University of Pennsylvania employing the multiplex xMAP Luminex platform (Luminex Corp., Austin, TX) and INNO-BIA AlzBio3 immunoassay reagents (Innogenetics, Ghent, Belgium; research use only) (Shaw et al., 2009). Participants were classified according to the ATN framework using previously established ADNI cutoffs derived from this assay platform (Aisen et al., 2010; Jack et al., 2024). A+ was defined as Aβ_42_ < 976.6 pg/ml, T+ as P-tau181 > 21.8 pg/ml, and N+ as T-tau > 245 pg/ml. These cutoffs were determined within the ADNI cohort using the same Luminex assay and are therefore assay-specific.

### 2.3 Cognitive assessments

Cognitive assessments included the MMSE to evaluate general cognition, as well as the composite scores for memory (ADNI_MEM), language (ADNI_LAN), visuospatial (ADNI_VS) and executive functioning (ADNI_EF) to assess specific cognitive domains. For all the above tests, higher scores indicate better cognitive function.

### 2.4 Neuroimaging

Structural T1-weighted MRI scans were acquired according to standardized ADNI MRI protocols on 1.5-T and 3.0-T scanners across participating sites. Detailed acquisition parameters for each ADNI phase have been described previously and are available on the ADNI website. MRI images underwent preprocessing and volumetric segmentation using FreeSurfer (v4.3 or v5.1) according to ADNI procedures. Post-quality control (QC) included visual inspection by trained raters, exclusion of gross segmentation failures and extreme outliers (e.g., >±4 SD). Regional brain volumes were normalized by intracranial volume (ICV) to account for individual differences in head size, and ICV was additionally included as a covariate in all MRI related analyses. To reduce variability arising from scanner type, field strength, imaging site, and FreeSurfer version, regional volumes were harmonized using the ComBat method while preserving biological covariates (age, sex, education, *APOE* ε4 status, and baseline diagnosis). The imaging outcomes included global brain volume and the following regions of interest (ROIs): hippocampus, entorhinal cortex, middle temporal gyrus, fusiform gyrus, and ventricles. These ROIs were selected based on their established vulnerability in AD related neurodegeneration.

### 2.5 Statistic analyses

Comparisons of baseline characteristics across diagnostic categories were conducted with *t*-tests or Wilcoxon rank-sum tests for continuous data and chi-square analyses for categorical factors. CSF biomarker data that did not follow a normal distribution (*P* < 0.05) were log10-transformed to approximate normality and subsequently standardized to z-score for downstream analyses.

First, group differences in CSF biomarkers related to endothelial dysfunction across ATN pathological categories were examined using analysis of covariance (ANCOVA).

Second, cross-sectional associations between CSF biomarkers related to endothelial dysfunction and AD core biomarkers, cognitive performance, and brain volumes were assessed using multivariable linear regression (MLR) models. Longitudinal associations with changes in AD biomarkers, cognition, and brain volumes were evaluated using linear mixed-effects (LME) models. Time was defined as years from baseline. An interaction term between baseline biomarker level and time (biomarker × time) was included to assess whether baseline biomarker levels were associated with differential rates of longitudinal change.

Third, Cox proportional hazards models were used to investigate associations between baseline biomarkers related to endothelial dysfunction and incident AD. Biomarkers were modeled continuously (per SD increase), and follow-up time was calculated from baseline to the first adjudicated diagnosis of AD dementia or the last available visit. The proportional hazards assumption was assessed using Schoenfeld residuals. To further evaluate the predictive utility and predictive performance of MMP-10 for incident AD, 3-year time-dependent ROC analyses were performed using nested Cox prediction models (Model 1: included clinical covariates consistent with the main Cox regression analyses, including age, sex, education, *APOE* ε4 status, hypertension, diabetes, dyslipidemia, smoking status, BMI, and cardiovascular disease; Model 2: additionally included CSF MMP-10; Model 3: included clinical covariates and Aβ_42_; Model 4: additionally included CSF MMP-10 on top of Model 3). The 3-years area under the curve (AUC) was calculated to assess model discrimination, and changes in AUC were used to evaluate the incremental predictive value of MMP-10.

Finally, mediation analyses were performed to investigate whether AD core biomarkers (Aβ_42_, P-tau, and T-tau) statistically accounted for the associations between these biomarkers and cognition. Indirect effects were estimated using 10,000 bootstrap resamples to obtain bias-corrected 95% confidence intervals.

All models were adjusted for age, sex, years of education, *APOE* ε4 carrier status, hypertension, diabetes, dyslipidemia, smoking status, body mass index (BMI), and cardiovascular disease. Intracranial volume (ICV) was additionally included in analyses involving brain volume outcomes.

Tolerance statistics, variance inflation factors (VIFs), and Pearson’s correlation coefficients were used to assess multicollinearity, and no major violations were identified. To account for multiple comparisons arising from testing eight biomarkers across multiple outcomes and models, false discovery rate (FDR) correction was applied within each outcome domain. All reported *P*-values in the main analyses were FDR-adjusted *P*-values. Because all continuous variables included in the regression models were standardized before analysis, the reported β coefficients represent standardized effect estimates, allowing direct comparison of effect sizes across biomarkers and outcomes. Statistical analyses and figure generation were performed using RStudio (version 4.2.1) and GraphPad Prism (version 9.4.2).

## 3 Results

### 3.1 Characteristics of participants

The characteristics of the non-demented participants (137 CN and 307 MCI) were demonstrated in Table 1. The average age of participants was 71.3 years; 171 participants were male. MCI participants had higher P-tau, T-tau and MMP-10, lower Aβ_42_ levels, MMSE, ADNI_MEM, hippocampal volume and entorhinal volume (*P* < 0.05) than CN participants. No significant differences were found between the two diagnostic groups in sex, educational levels, hypertension, diabetes, dyslipidemia, smoking status, BMI, and cardiovascular disease, or scores on ADNI_EF, ADNI_LAN, and ADNI_VS.

**TABLE 1:** Clinical characteristics of participants in the current study.

Characteristics	CN	MCI	*P*
Number	137	307	
Age (y)	73.94 ± 5.51	71.32 ± 7.50	**<0.001**
Gender (female/male)	64/73	136/171	0.333
Education (y), median [IQR]	16.00 [14.00, 18.00]	16.00 [14.00, 18.00]	0.125
*APOE ε4* carriers (0/1)	102/39	141/166	**<0.001**
Cardiovascular disease (0/1)	129/8	285/22	0.607
Diabetes (0/1)	127/10	277/30	0.401
Hypertension (0/1)	79/58	197/110	0.192
Dyslipidemia (0/1)	79/58	155/152	0.162
Smoking status (0/1)	105/32	251/56	0.212
BMI (kg/m^2^)	26.28 [24.13, 29.26]	26.37 [24.11, 29.37]	0.825
Clinical assessments			
MMSE, (median [IQR])	29.00 [29.00, 30.00]	28.00 [26.00, 29.00]	**<0.001**
ADNI_MEM	0.58 [0.01, 1.15]	0.37 [−0.13, 0.89]	**0.022**
ADNI_EF	0.67 [−0.16, 1.23]	0.38 [−0.17, 0.94]	0.060
ADNI_LAN	0.49 ± 0.91	0.38 ± 0.74	0.227
ADNI_VS	−0.08 [−0.43, 0.74]	−0.08 [−0.43, 0.74]	0.707
MRI measures			
Ventricles (mm^3^)	31217.50 [18998.25, 39282.25]	32600.50 [21697.25, 49359.00]	0.059
Hippocampus (mm^3^)	7463.75 ± 834.59	6865.13 ± 1167.71	**<0.001**
Whole brain (mm^3^)	1036641.54 ± 105137.86	1036529.32 ± 110156.92	0.992
Entorhinal (mm^3^)	3873.94 ± 640.45	3527.29 ± 727.35	**<0.001**
Fusiform (mm^3^)	18218.50 [16481.00, 19838.00]	17741.50 [15813.25, 19695.75]	0.088
Mid temporal lobe (mm^3^)	20324.85 ± 2612.79	19949.90 ± 3053.78	0.214
Biomarkers			
CSF Aβ_42_ (pg/mL)	1332.00 [831.30, 1843.00]	837.70 [633.05, 1288.00]	**<0.001**
CSF P-tau (pg/mL)	19.39 [15.88, 25.94]	24.99 [17.82, 36.45]	**<0.001**
CSF T-tau (pg/mL)	213.90 [179.60, 279.30]	263.80 [199.10, 360.35]	**<0.001**
CSF PDGFRβ (RFU)	332.90 [296.50, 370.60]	331.90 [310.00, 367.25]	0.738
CSF Osteopontin (RFU)	28553.60 [25364.50, 31759.40]	28837.90 [24998.90, 33141.05]	0.493
CSF VCAM-1 (RFU)	1077.00 [924.80, 1240.00]	1085.70 [931.35, 1295.25]	0.473
CSF E-selectin (RFU)	852.50 [689.80, 1086.20]	846.80 [655.00, 1103.35]	0.722
CSF MMP-2 (RFU)	7528.10 [6736.80, 8702.30]	7216.60 [6520.70, 7946.40]	**0.010**
CSF MMP-10 (RFU)	118.80 [111.30, 126.90]	126.10 [115.10, 140.60]	**<0.001**
CSF MMP-3 (RFU)	2948.70 [2565.90, 3434.20]	2848.40 [2536.30, 3232.20]	0.256
CSF MMP-9/TIMP-1	0.01 [0.00, 0.01]	0.01 [0.00, 0.01]	0.426
MMP-12 (RFU)	121.50 [112.40, 153.60]	121.30 [110.70, 143.60]	0.616
Periostin (RFU)	377.60 [311.80, 462.10]	346.40 [286.55, 432.90]	0.095

Categorical variables are reported as numbers and percentages; continuous variables are reported as means ± SDs or median [first quartile to third quartile] as appropriate. ADNI, Alzheimer’s disease Neuroimaging Initiative; *APOE ε4*, apolipoprotein E4; Aβ_42_, amyloid β peptide 42; PDGFRβ, platelet-derived growth factor β; VCAM-1, vascular cell adhesion molecule-1; MMP-2, matrix metalloproteinase 2; MMP-10, matrix metalloproteinase 10; MMP-3, matrix metalloproteinase 3; MMP-12, matrix metalloproteinase-12; MMP-9/TIMP-1, matrix metalloproteinase 9/tissue inhibitors of metalloproteinase-1; MMP-12, matrix metalloproteinase 12; CSF, cerebrospinal fluid; EF, executive function; LAN, language; MEM, memory function; MMSE, Mini-Mental State Examination; P-tau, phosphorylated tau; T-tau, total tau; BMI, body mass index. Bold values indicate statistical significance (*P* < 0.05).

### 3.2 Group differences in CSF biomarkers related to endothelial dysfunction across ATN categories

In the A+/A− groups, VCAM-1 was the only biomarker that differed significantly between groups (*P* = 0.024) (Figure 1A). In contrast, more pronounced differences were observed in the tau (T+/T−) and neurodegeneration (N+/N−) groups. Specifically, PDGFRβ, Osteopontin, VCAM-1, and MMP-10 levels were significantly higher in both the T+ and N+ groups than their respective T− and N− groups (all *P* ≤ 0.031) (Figures 1B–I).

**FIGURE 1:**
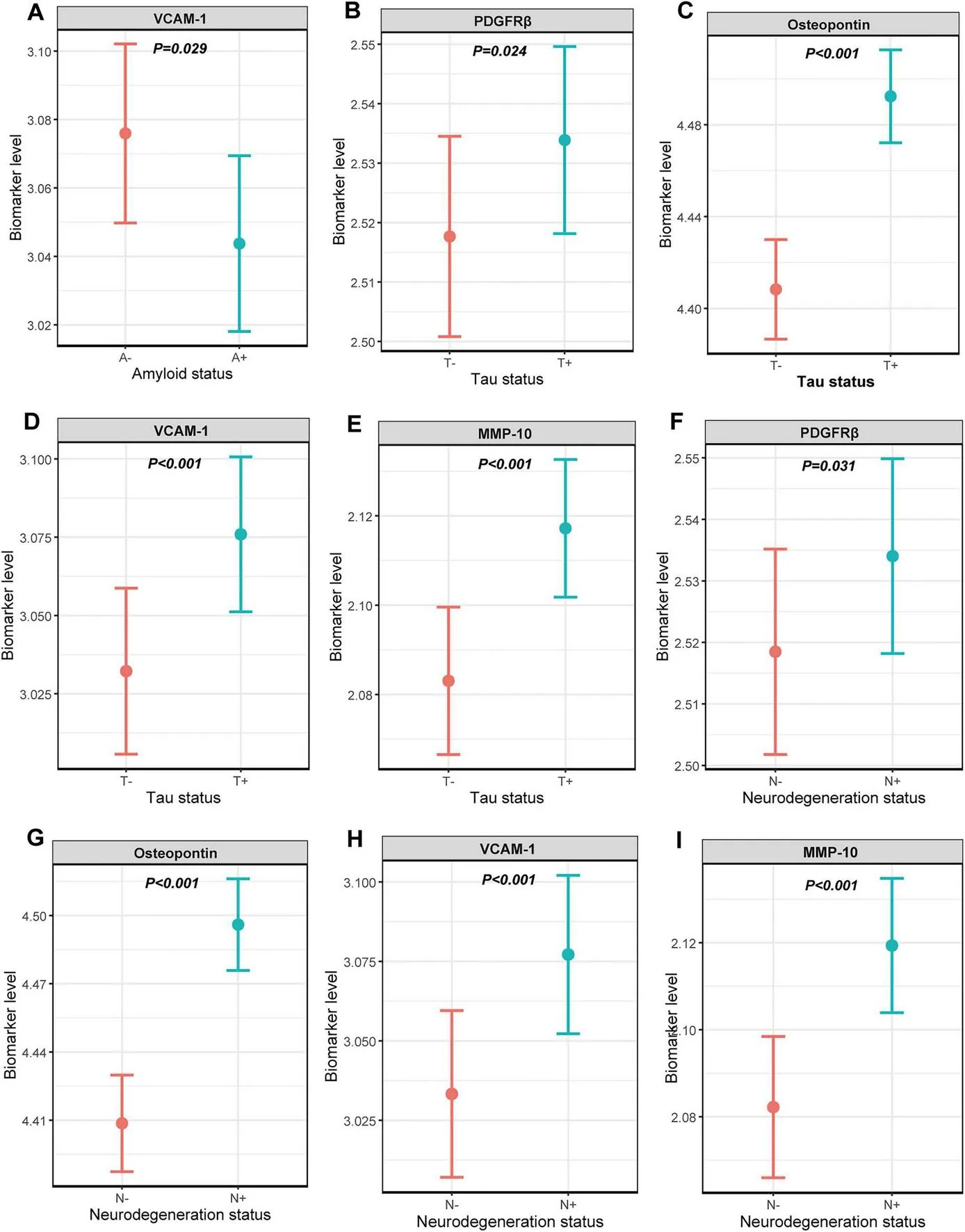
Group differences in CSF biomarkers related to endothelial dysfunction across ATN categories. PDGFRβ, platelet-derived growth factor β; VCAM-1, vascular cell adhesion molecule-1; MMP-10, matrix metalloproteinase 10; MMP-9/TIMP-1, matrix metalloproteinase 9/tissue inhibitors of metalloproteinase-1; A–, Aβ_42_ negative; A+, Aβ_42_ positive; T–, P-tau negative; T+, P-tau positive; N–, T-tau negative; N+, T-tau positive. **(A)** The group differences of VCAM-1 between different A status. **(B–E)** The group differences of Osteopontin, VCAM-1, MMP-10 and PDGFRβ between different T status. **(F–I)** The group differences of Osteopontin, VCAM-1, MMP-10 and PDGFRβ between different N status. *P*-values were obtained from ANCOVA models adjusted for age, sex, education, *APOE* ε4 status, hypertension, diabetes, dyslipidemia, smoking status, BMI, and cardiovascular disease, and were FDR-adjusted.

### 3.3 The associations between CSF biomarkers related to endothelial dysfunction and AD core biomarkers

In cross-sectional analyses, only two biomarkers related to endothelial dysfunction showed significant associations with Aβ pathology. Specially, higher MMP-10 levels were associated with lower Aβ_42_ (β = −0.150, *P* = 0.014), higher VCAM-1 levels were associated with higher Aβ_42_ (β = 0.222, *P* < 0.001) (Figure 2A). Most biomarkers showed significant associations with tau-related pathologies. Specifically, higher MMP-10 (P-tau: β = 0.382, *P* < 0.001; T-tau: β = 0.378, *P* < 0.001), MMP-3 (P-tau: β = 0.257, *P* < 0.001; T-tau: β = 0.250, *P* < 0.001), PDGFRβ (P-tau: β = 0.221, *P* < 0.001; T-tau: β = 0.240, *P* < 0.001), Osteopontin (P-tau: β = 0.577, *P* < 0.001; T-tau: β = 0.579, *P* < 0.001), VCAM-1 (P-tau: β = 0.153, *P* = 0.009; T-tau: β = 0.181, *P* = 0.001) and MMP-9/TIMP-1 ratio (P-tau: β = 0.203, *P* < 0.001; T-tau: β = 0.218, *P* < 0.001) were associated with higher levels of P-tau and T-tau (Figures 2B, C). Higher E-selectin levels were associated with lower P-tau (β = −0.130, *P* = 0.046).

**FIGURE 2:**
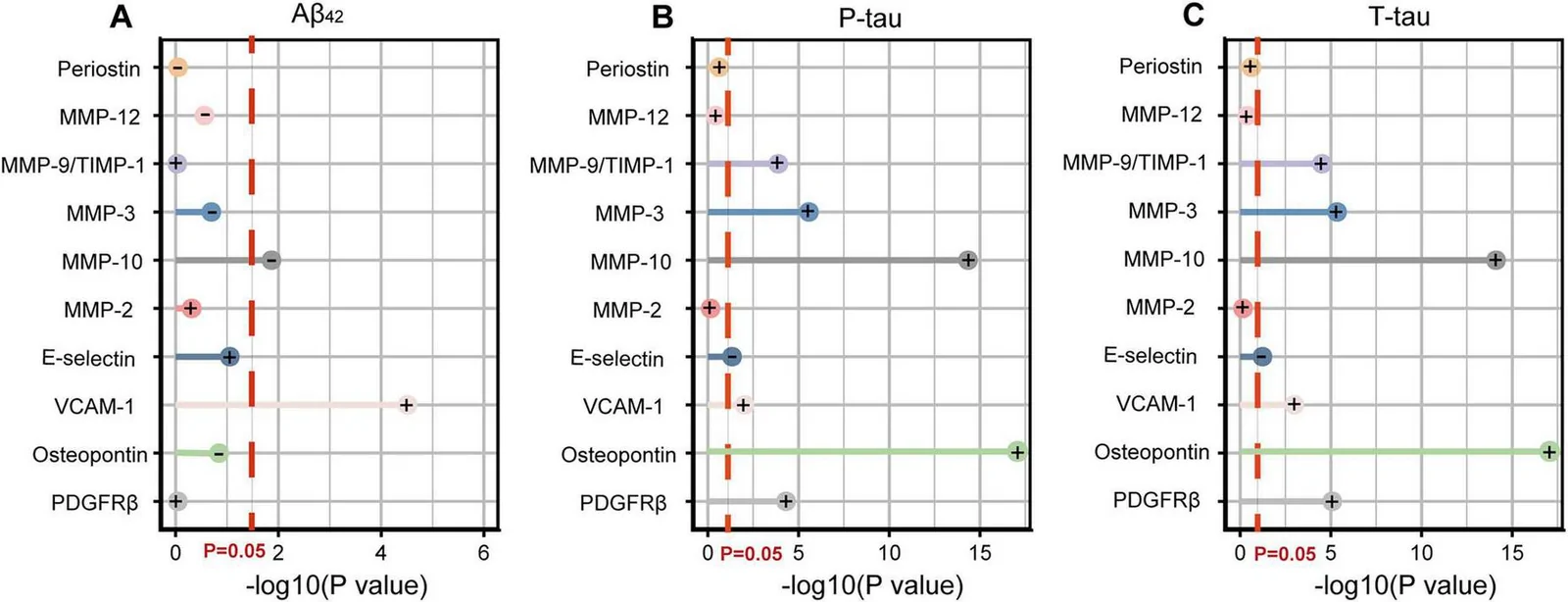
The associations between CSF biomarkers related to endothelial dysfunction with AD core biomarkers. Aβ_42_, amyloid β peptide 42; P-tau, phosphorylated tau; T-tau, total tau; PDGFRβ, platelet-derived growth factor β; VCAM-1, vascular cell adhesion molecule-1; MMP-2, matrix metalloproteinase 2; MMP-10, matrix metalloproteinase 10; MMP-3, matrix metalloproteinase 3; MMP-12, matrix metalloproteinase-12; MMP-9/TIMP-1, matrix metalloproteinase 9/tissue inhibitors of metalloproteinase-1. **(A–C)** Multiple linear regression models were used to examine the associations between CSF biomarkers related to endothelial dysfunction and CSF Aβ_42_, CSF p-tau, and CSF t-tau, adjusting for age, sex, education, *APOE* ε4 status, hypertension, diabetes, dyslipidemia, smoking status, BMI, and cardiovascular disease. Circles indicate the direction of association: “+” denotes a positive association (β > 0), and “–” denotes a negative association (β < 0). Statistical significance is indicated within each panel as specified. *P*-values are FDR-adjusted.

In longitudinal analyses, baseline levels of CSF biomarkers related to endothelial dysfunction were not associated with subsequent changes in AD core biomarkers over time, as no significant biomarker × time interactions were observed (Supplementary Table 2).

### 3.4 The associations between CSF biomarkers related to endothelial dysfunction and cognition

In cross-sectional analyses, higher MMP-10 levels were significantly associated with poorer cognitive performance across multiple domains (ADNI_MEM: β = −0.264, *P* < 0.001; ADNI_EF: β = −0.211, *P* < 0.001; ADNI_LAN: β = −0.215, *P* < 0.001; ADNI_VS: β = −0.107, *P* = 0.007) (Figures 3A–E). Moreover, higher MMP-3 and Osteopontin levels were associated with poorer memory performance (ADNI_MEM; MMP-3: β = −0.139, *P* < 0.001; Osteopontin: β = −0.096, *P* = 0.047), whereas higher MMP-12 levels were associated with poorer visuospatial performance (ADNI_VS: β = −0.149, *P* < 0.001) (Figures 3A, B). No significant associations were observed for the other biomarkers.

**FIGURE 3:**
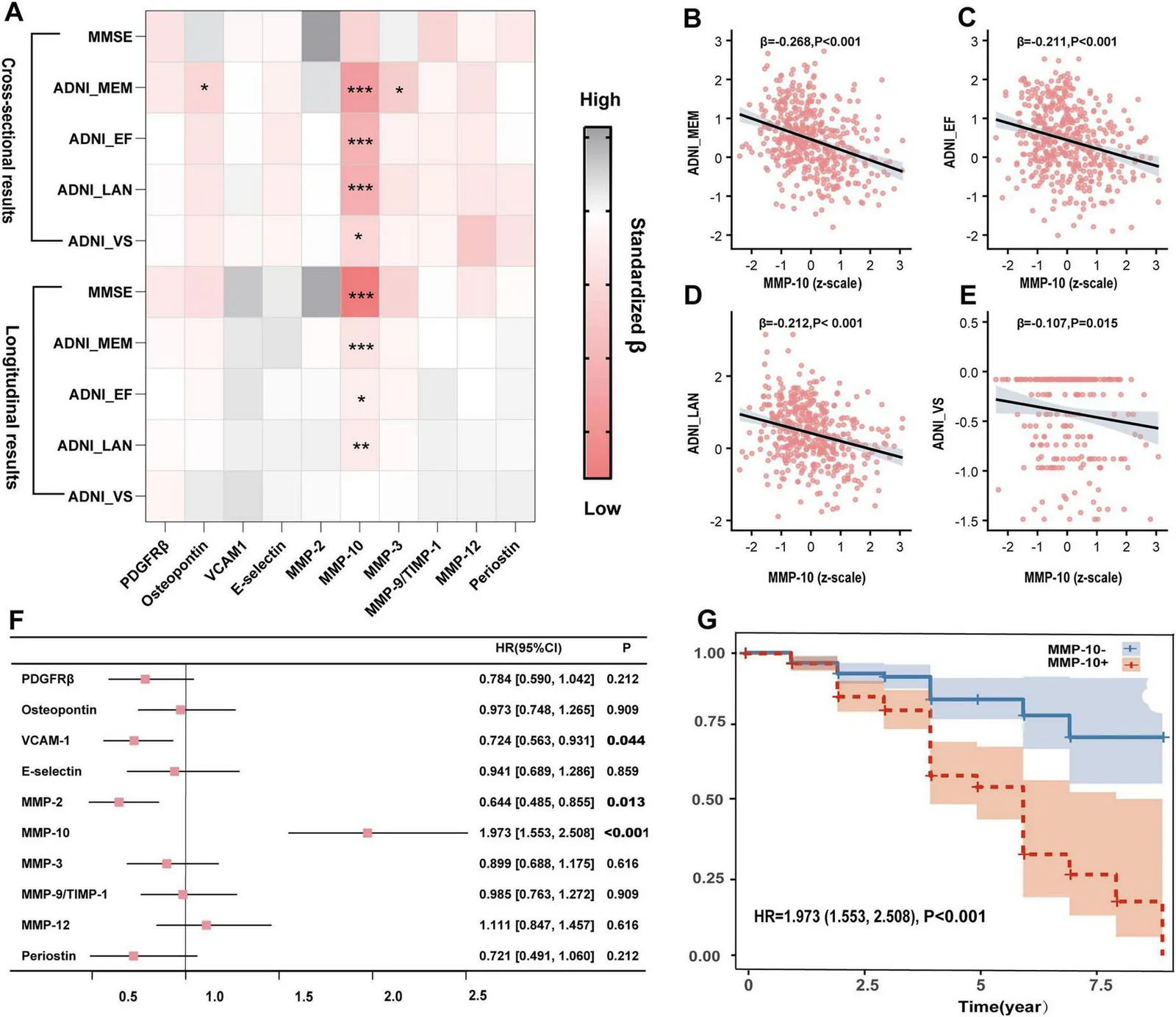
The associations between CSF biomarkers related to endothelial dysfunction and cognition. ADNI, Alzheimer’s disease Neuroimaging Initiative; PDGFRβ, platelet-derived growth factor β; VCAM-1, vascular cell adhesion molecule-1; MMP-2, matrix metalloproteinase 2; MMP-10, matrix metalloproteinase 10; MMP-3, matrix metalloproteinase 3; MMP-12, matrix metalloproteinase-12; MMP-9/TIMP-1, matrix metalloproteinase 9/tissue inhibitors of metalloproteinase-1; CSF, cerebrospinal fluid; EF, executive function; LAN, language; MEM, memory function; MMSE, Mini-Mental State Examination; HR, hazard ratio. **(A)** Multiple linear regression models were used to examine associations of CSF biomarkers related to endothelial dysfunction with cognition, and LME showed the relationships of each baseline CSF biomarkers related to endothelial dysfunction with longitudinal changes in cognitive adjusting for age, sex, education, *APOE* ε4 status, hypertension, diabetes, dyslipidemia, smoking status, BMI, and cardiovascular disease. Significant results were marked with asterisks. *P < 0.05, **P < 0.01, and ***P < 0.001. **(B–E)** Scatter plot showed the association between CSF MMP-10 levels and cognitive performance. **(F)** The Cox proportional hazards model revealed the risk of incident AD dementia during the follow-up of eight biomarkers related to endothelial dysfunction. **(G)** The K-M curve showed the risk of incident AD dementiain MMP-10. *P*-values are FDR-adjusted.

In longitudinal analyses, consistent with the cross-sectional findings, higher baseline MMP-10 levels were associated with faster cognitive decline across multiple domains over time, including global cognition (MMSE: β = −0.363, *P* < 0.001), memory (ADNI_MEM: β = −0.073, *P* < 0.001), executive function (ADNI_EF: β = −0.059, *P* = 0.022), language (ADNI_LAN: β = −0.059, *P* = 0.001). However, we did not find any significant associations between the other biomarkers and cognitive changes (Supplementary Table 3).

### 3.5 Associations between CSF biomarkers related to endothelial dysfunction and the risk of incident AD

In the Cox proportional hazards model, higher MMP-2 and VCAM-1 levels were associated with lower risks of incident AD, corresponding to 35.6% and 27.6% lower risks per 1-SD increase, respectively (MMP-2: HR = 0.644, 95% CI = 0.485–0.855, *P* = 0.013; VCAM-1: HR = 0.724, 95% CI = 0.563–0.931, *P* = 0.044). In contrast, each 1-SD increase in CSF MMP-10 was associated with an approximately 97.3% higher risk of incident AD dementia (HR = 1.973, 95% CI = 1.553–2.508, *P* < 0.001), supporting a clinically meaningful prognostic association (Figures 3F, G). No significant associations with the incident AD dementia were found for the other biomarkers.

To further assess the predictive value of MMP-10, we performed 3-year time-dependent ROC analyses using nested Cox prediction models. The clinical model yielded an AUC of 0.650. Addition of MMP-10 increased the AUC to 0.721, corresponding to a ΔAUC of 0.071. The model including clinical covariates and AD core biomarkers showed an AUC of 0.761, and further addition of MMP-10 increased the AUC to 0.793, corresponding to a ΔAUC of 0.032 (Supplementary Figure 1).

### 3.6 The associations between CSF biomarkers related to endothelial dysfunction and brain volumes

In cross-sectional analyses, higher VCAM-1 levels were associated with larger whole-brain volume (β = 0.079, *P* = 0.018). Higher MMP-3 levels were associated with smaller fusiform volume (β = −0.145, *P* = 0.008), whereas higher POSTN levels were associated with smaller ventricular volume (β = −0.106, *P* = 0.042) (Figure 4A).

**FIGURE 4:**
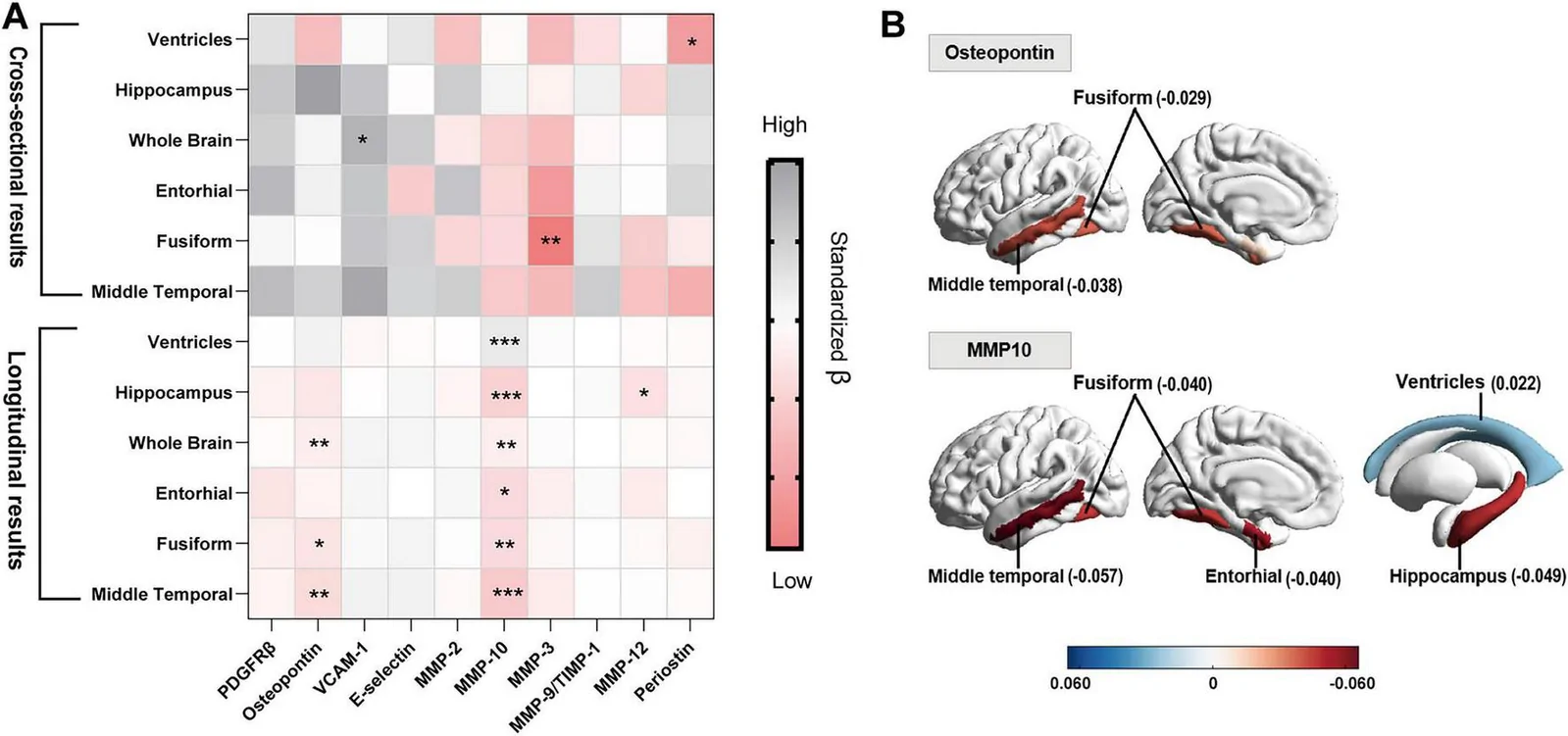
The associations between CSF biomarkers related to endothelial dysfunction and brain volumes. PDGFRβ, platelet-derived growth factor β; VCAM-1, vascular cell adhesion molecule-1; MMP-2, matrix metalloproteinase 2; MMP-10, matrix metalloproteinase 10; MMP-3, matrix metalloproteinase 3; MMP-9/TIMP-1, matrix metalloproteinase 9/tissue inhibitors of metalloproteinase-1; **(A)** Multiple linear regression models were used to examine associations of CSF biomarkers related to endothelial dysfunction with brain volume, and LME showed the relationships of each baseline CSF biomarkers related to endothelial dysfunction with longitudinal changes in brain volume adjusting for age, sex, education, *APOE* ε4 status, hypertension, diabetes, dyslipidemia, smoking status, BMI, and cardiovascular disease. Significant results were marked with asterisks. *P < 0.05, **P < 0.01, and ***P < 0.001. *P*-values are FDR-adjusted. **(B)** The figure showed the relationship between MMP-10, Osteopontin and different brain regions atrophy over time.

Longitudinal analyses revealed significant associations between biomarkers related to endothelial dysfunction and progressive brain atrophy over time (Figures 4A, B). It was noteworthy that higher baseline MMP-10 levels were consistently associated with whole brain atrophy and all regional volume loss (whole brain: β = −0.019, *P* = 0.002; ventricular: β = 0.022, *P* < 0.001; hippocampus: β = −0.045, *P* < 0.001; middle temporal: β = −0.052, *P* < 0.001; entorhinal cortex: β = −0.039, *P* = 0.047; fusiform gyrus: β = −0.039, *P* = 0.002). Similarly, higher Osteopontin levels were associated with greater atrophy in the fusiform, middle temporal, and whole brain (all *p* ≤ 0.046) (Figure 4B and Supplementary Table 4).

### 3.7 Mediation analyses

We summarized the relationships of the CSF biomarkers related to endothelial dysfunction with AD core biomarkers, brain volume, cognitive function, and risk of incident AD. Among them, MMP-10 was the only marker that showed significant associations across all four domains examined. The other biomarkers were associated with selected outcomes but did not show consistent associations across all domains. Based on these findings, biomarkers that were significantly associated to both AD core pathology and cognitive function were included in subsequent mediation analyses (Figure 5A).

**FIGURE 5:**
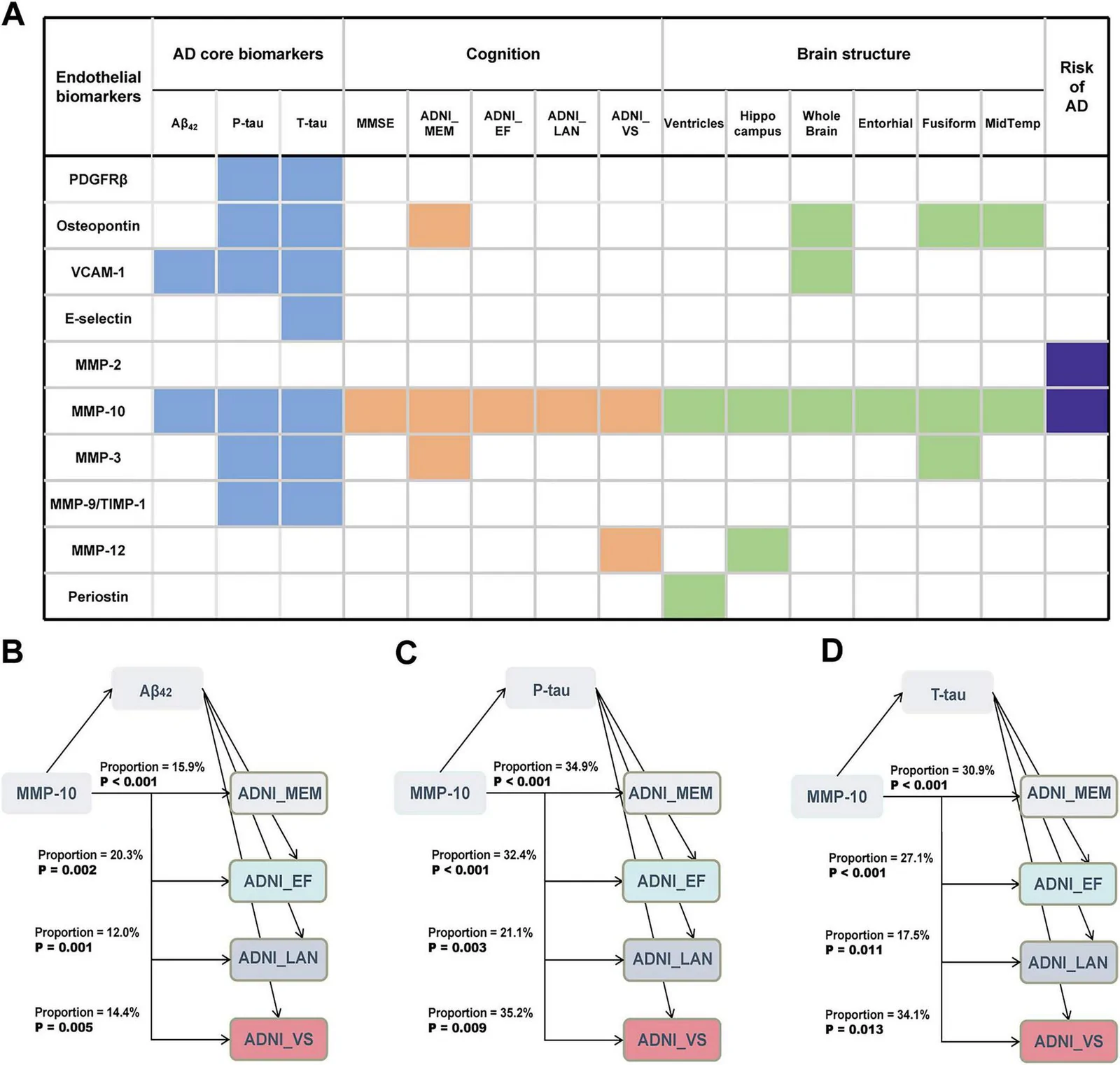
Summary of associations between CSF biomarkers related to endothelial dysfunction and AD-related outcomes and mediation analyses. MMP-10, matrix metalloproteinase 10; MMP-3, matrix metalloproteinase 3; CSF, cerebrospinal fluid; EF, executive function; LAN, language; MEM, memory function; MMSE, Mini-Mental State Examination; P-tau, phosphorylated tau; T-tau, total tau. **(A)** This schematic overview illustrates the associations of ten endothelial dysfunction–related CSF biomarkers with core AD pathological biomarkers, cognitive performance, brain structural measures, and the risk of incident AD dementia during follow-up. Each colored square indicates a statistically significant association between the corresponding biomarker and outcome. Different colors denote outcome categories: core AD biomarkers (blue), cognitive measures (orange), brain structural measures (green), and risk of incident AD dementia (purple). **(B–D)** The relationship between MMP-10 and cognition was mediated by Aβ42, P-tau, T-tau. *P*-values were obtained from mediation models adjusted for age, sex, education, *APOE* ε4 status, hypertension, diabetes, dyslipidemia, smoking status, BMI, and cardiovascular disease, and were FDR-adjusted.

Specifically, mediation analyses suggested that Aβ_42_, P-tau, and T-tau statistically accounted for part of the associations between MMP-10 and cognitive performance, with estimated mediation proportions ranging from 12.0% to 20.3%, 21.1% to 34.9%, and 17.5% to 34.1%, respectively (Figures 5B–D and Supplementary Table 5).

## 4 Discussion

The study comprehensively examined the relationships between CSF biomarkers related to endothelial dysfunction and AD pathologies, cognition, brain volumes and AD risk in non-demented adults. Two main findings emerged. First, among the biomarkers examined, MMP-10 showed the most consistent associations with AD. Second, endothelial dysfunction-related biomarkers were more consistently associated with CSF tau biomarkers than with Aβ_42_. Participants in the T+ and N+ groups showed more pronounced differences in these biomarkers than those in the A+ group, and cross-sectional mediation analyses suggested that CSF tau biomarkers partially accounted for the associations between endothelial dysfunction and cognition. These findings suggest that endothelial dysfunction related processes may be preferentially linked to tau related neurodegenerative changes during the non-demented stages of AD.

First of all, among these biomarkers related to endothelial dysfunction, MMP-10 was more closely related to AD. Higher CSF MMP-10 levels were associated with lower CSF Aβ_42_ and higher CSF tau biomarkers, as well as poorer cognitive performance. These findings are in line with a previous study in 256 participants reporting associations between MMP-10 and both amyloid and tau biomarkers, as well as longitudinal cognitive decline (Aksnes et al., 2023b). Previous studies did not find MMP-10 had any association with brain volumes (Abe et al., 2020; Aksnes et al., 2023a), but our study found that higher baseline CSF MMP-10 were associated with greater longitudinal reductions in the whole brain volume, as well as entorhinal, hippocampal and middle temporal lobe volumes. Differences in cohort characteristics, disease stage distribution, adjustment strategies, and imaging methodology may partly explain these discrepancies. Taken together, these results highlight MMP-10 as a potentially important biomarker related to endothelial dysfunction that may bridge vascular injury, AD core pathology, cognitive decline, and brain atrophy.

In addition to MMP-10, other MMPs showed more selective associations. MMP-3 was primarily associated with CSF tau biomarkers and cognitive performance, consistent with previous human studies linking MMP-3 to neuroinflammatory processes (Aksnes et al., 2023b; Peng et al., 2015). We did not observe significant associations with Aβ_42_ in our cohort. However, experimental studies in animal models have reported elevated MMP-3 expression in Tg2576 mice and in the McGill-R-Thy1-APP transgenic rat model, where it was observed in association with amyloid plaque pathology (Bluhm et al., 2022; Pentz et al., 2021). These discrepancies may reflect differences between central tissue expression in transgenic models and CSF biomarker levels in humans, as well as variations in disease stage, species-specific biology, and methodological approaches. In contrast, MMP-2 demonstrated a distinct pattern, with higher levels associated with lower risk of incident AD dementia, further highlighting heterogeneity across MMP subtypes. MMP-12 was selectively associated with cognitive performance in our study. This observation is in line with experimental evidence showing that MMP-12 knockdown reduced post-ischemic infarct volume and improved motor and cognitive recovery, suggesting a potential role of MMP-12 in vascular injury-related neural dysfunction (Arruri et al., 2022). The MMP-9/TIMP-1 ratio was associated with CSF tau biomarkers but not with structural brain measures, further indicating variability across MMP subtypes. Taken together, these findings suggest that although multiple MMPs are related to AD-related processes, MMP-10 exhibited the most consistent and robust associations across domains.

Apart from MMPs, our study also found that PDGFRβ, Osteopontin, E-selectin, VCAM-1 and POSTN were associated with AD. PDGFRβ was associated with CSF tau biomarkers but not with CSF Aβ_42_, consistent with previous human studies (Haghdel et al., 2024; Vrillon et al., 2025). Osteopontin levels were associated with brain structural measures and cognitive performance in our cohort, consistent with findings from previous population-based and experimental animal studies (Azizan et al., 2025; Karasalih et al., 2025; Qiu et al., 2023; Yu et al., 2024). E-selectin showed associations with CSF tau biomarkers in our study, whereas previous investigations did not consistently observe such relationships (Li et al., 2015). Differences in disease stage composition, biomarker modality, and analytical adjustments may partly explain this discrepancy. VCAM-1 was positively associated with both CSF Aβ_42_ and tau levels in our cohort. The concordant direction of these associations may reflect a generalized state of endothelial activation and vascular inflammation that influences the dynamics of both amyloid and tau metabolism rather than a direct effect on a single pathological pathway (Drake et al., 2021; Reglero-Real et al., 2016). As CSF Aβ_42_ reflects soluble peptide levels rather than deposited plaque burden, endothelial dysfunction may be associated with changes in BBB permeability or clearance mechanisms, which in turn may relate to parallel alterations in CSF Aβ_42_ and tau levels (Hartmann et al., 2026; Nation et al., 2019). POSTN showed a selective association with brain structure, with higher CSF POSTN levels related to smaller ventricular volume. Although direct evidence linking CSF POSTN to ventricular volume in AD remains limited, experimental studies suggest that POSTN may be involved in brain injury-response and reparative processes, including neural stem cell proliferation and differentiation after hypoxic-ischemic injury (Ma et al., 2015). Taken together, these findings suggest that these biomarkers are linked to AD-related changes, but their associations vary in strength and domain specificity.

Furthermore, the study results suggested a more consistent association between endothelial dysfunction and tau biomarkers than with Aβ pathology. Compromise of BBB integrity have been associated with tau pathology (Huang et al., 2025). BBB permeability has been shown to correlate with tau pathology in both preclinical and early symptomatic stages, and vascular dysfunction may be involved in processes related to tau accumulation or spread through neuroinflammatory activation, perivascular damage, and impaired clearance mechanisms (Chen et al., 2025; Sanchez et al., 2025; Vollhardt et al., 2025). White matter hyperintensity (WMH), a pathological manifestation of vascular injury, has also been linked to tau pathology rather than Aβ pathology (Parent et al., 2026; Rust et al., 2025). Moreover, several endothelial dysfunction biomarkers were associated with brain atrophy and cognitive performance, consistent with prior studies showing that tau pathology broadly correlates with cortical atrophy and cognitive decline (Digma et al., 2019; Tanner et al., 2022). Importantly, the stronger associations with tau biomarkers should not be interpreted solely as evidence of AD-specific tau mechanisms. CSF tau, particularly T-tau, may also reflect generalized neurodegeneration and neuronal injury beyond AD-specific pathological processes (Blennow and Zetterberg, 2018; Palmqvist et al., 2020). Disease-stage effects may further contribute to these findings, as amyloid pathology is thought to emerge early and often reaches a relative plateau before symptom onset, whereas tau-related changes remain more dynamic during the preclinical and prodromal stages represented in our cohort (Jack et al., 2018). Additionally, several biomarkers examined in this study, including MMPs and Osteopontin, are involved in inflammatory and extracellular matrix remodeling pathways. Therefore, their associations with tau biomarkers may partly reflect inflammation-related neuronal injury and tissue damage rather than direct effects on tau pathology itself (Leng and Edison, 2021; Pascoal et al., 2021). Consequently, the stronger relationships observed between endothelial dysfunction-related biomarkers and tau measures may reflect a combination of vascular dysfunction, neurodegeneration, disease-stage characteristics, and inflammatory processes rather than a single AD-specific pathway. Importantly, the relationship between endothelial dysfunction-related processes and AD pathology may be bidirectional. Neurodegenerative processes, particularly tau-related pathology, may contribute to BBB disruption and endothelial dysfunction through neuroinflammation, perivascular injury, and impaired vascular integrity (Montagne et al., 2015; Nation et al., 2019; Sweeney et al., 2018). Therefore, some observed associations between CSF biomarkers related to endothelial dysfunction and tau biomarkers, cognitive decline, or brain atrophy may partly reflect downstream vascular changes secondary to neurodegeneration, rather than solely causal effects of vascular dysfunction. Future longitudinal studies with repeated vascular and AD biomarker assessments are needed to clarify the temporal ordering and directionality of these associations.

This study is subject to certain limitations. First, the modest sample size and incomplete PET imaging data may limit the generalizability of our findings and the ability to fully explore associations with AD pathologies. Second, as most of the findings were derived from cross-sectional analyses, longitudinal studies with repeated biomarker assessments are needed to clarify temporal dynamics, underlying mechanisms, and potential causal relationships. Third, several of the biomarkers examined in this study are not exclusively specific to endothelial cells, their biological interpretation should be made cautiously. Fourth, the mediation analyses should be interpreted cautiously because the cross-sectional design cannot establish temporal ordering, and unmeasured confounding across mediation pathways cannot be excluded. Thus, these results should be viewed as statistical decompositions of associations rather than evidence of causal pathways. Finally, given the observational and predominantly cross-sectional design, we cannot exclude the possibility of reverse causation, whereby neurodegenerative or tau related pathology contributes to endothelial dysfunction related processes. Therefore, our findings should be interpreted as statistical associations rather than evidence of unidirectional causal pathways.

In conclusion, endothelial dysfunction biomarkers were more consistently associated with tau biomarkers than with Aβ biomarkers in AD. In addition, among these biomarkers, MMP-10 showed relatively consistent associations across pathological and clinical measures, indicating that it may merit further investigation. Overall, our results point to a potential role of CSF biomarkers related to endothelial dysfunction in the vascular contribution to AD and highlight the importance of future longitudinal and mechanistic studies to better define their involvement in disease progression.

## Data Availability

Publicly available datasets were analyzed in this study. This data can be found here: https://adni.loni.usc.edu/.

## Footnotes

1
https://adni.loni.usc.edu
