# Supplementary material for: Associations between cerebrospinal fluid biomarkers related to endothelial dysfunction and Alzheimer’s disease
Source: Front Aging Neurosci. 2026 Sep 21;18:1744647. doi: 10.3389/fnagi.2026.1744647 (PMC13635297; doi:10.3389/fnagi.2026.1744647)
Supplement: Supplementary file 1 [file Data_Sheet_1.doc]

**Associations between cerebrospinal fluid biomarkers related to endothelial dysfunction and Alzheimer’s disease pathology, cognition, brain structure and risk of incident AD dementia**

### Supplementary material

**Content**

Table S1 The number of participants and repeated observations included in each linear mixed-effects model	1

Table S2 Associations of CSF biomarkers related to endothelial dysfunction with longitudinal changes in AD core biomarkers	2

Table S3 Associations of CSF biomarkers related to endothelial dysfunction with longitudinal changes in cognition	3

Table S4 Associations of CSF biomarkers related to endothelial dysfunction with longitudinal changes in brain volumes	4

Figure S1 Three-year time-dependent ROC curves for predicting incident AD dementia	5

Table S5 Mediating effects of CSF biomarkers related to endothelial dysfunction on the association between AD pathology and cognition in cognitively normal participants.	6

## Table S1 The number of participants and repeated observations included in each linear mixed-effects model

| Outcomes | N_participants | N_observations | Median follow-up, years |
| --- | --- | --- | --- |
| Aβ_42_ | 295 | 770 | 2.61 |
| P-tau | 294 | 765 | 2.60 |
| T-tau | 295 | 770 | 2.61 |
| MMSE | 295 | 770 | 2.61 |
| ADNI_MEM | 295 | 770 | 2.61 |
| ADNI_EF | 295 | 766 | 2.60 |
| ADNI_LAN | 295 | 770 | 2.61 |
| ADNI_VS | 295 | 770 | 2.61 |
| Ventricular | 292 | 675 | 2.31 |
| Hippocampus | 285 | 650 | 2.28 |
| Whole brain | 295 | 698 | 2.37 |
| Entorhinal | 279 | 593 | 2.13 |
| Fusiform | 279 | 593 | 2.13 |
| Middle temporal | 279 | 593 | 2.13 |

Note: This table summarizes the number of participants and repeated observations included in the linear mixed-effects models for each longitudinal outcome. N_participants indicates the number of individuals contributing at least one valid observation for the corresponding outcome, and N_observations indicates the total number of non-missing repeated measurements used in the model. Median follow-up was defined as the median interval from baseline to the last available assessment for each outcome. Differences in sample size across outcomes reflect differences in follow-up data availability.

## Table S2 Associations of CSF biomarkers related to endothelial dysfunction with longitudinal changes in AD core biomarkers

|  | Aβ_42_ | | | P-tau | | | T-tau | | |
| --- | --- | --- | --- | --- | --- | --- | --- | --- | --- |
|  | β | 95%CI | p value | β | 95%CI | p value | β | 95%CI | p value |
| PDGFRβ | 0.004 | (-0.011, 0.019) | 0.882 | 0.002 | (-0.009, 0.013) | 0.931 | 0.001 | (-0.010, 0.0128) | 0.940 |
| Osteopontin | -0.002 | (-0.017, 0.013) | 0.940 | 0.008 | (-0.003, 0.019) | 0.660 | 0.007 | (-0.005, 0.018) | 0.676 |
| VCAM-1 | 0.010 | (-0.005, 0.024) | 0.659 | 0.001 | (-0.010, 0.011) | 0.972 | -0.004 | (-0.015, 0.006) | 0.781 |
| E-selectin | 0.008 | (-0.006, 0.023) | 0.676 | -0.001 | (-0.011, 0.012) | 0.990 | -0.001 | (-0.012, 0.010) | 0.966 |
| MMP-2 | 0.016 | (0.002, 0.031) | 0.209 | -0.002 | (-0.0133, 0.0088) | 0.927 | -0.001 | (-0.011, 0.011) | 0.990 |
| MMP-10 | 0.002 | (-0.012, 0.017) | 0.939 | 0.002 | (-0.0087, 0.0127) | 0.927 | 0.011 | (-1e-04, 0.022) | 0.331 |
| MMP-3 | 0.009 | (-0.01, 0.024) | 0.675 | -0.003 | (-0.0147, 0.008) | 0.852 | -0.003 | (-0.014, 0.009) | 0.923 |
| MMP-9/TIMP-1 | -0.0046 | (-0.019, 0.010) | 0.852 | -0.008 | (-0.019, 0.002) | 0.545 | -0.013 | (-0.024, -0.002) | 0.175 |
| MMP-12 | 0.003 | (-0.012, 0.018) | 0.923 | -0.004 | (-0.015, 0.007) | 0.781 | -0.003 | (-0.014, 0.009) | 0.919 |
| Periostin | 0.003 | (-0.013, 0.020) | 0.927 | -0.002 | (-0.014, 0.010) | 0.937 | -0.002 | (-0.015, 0.010) | 0.927 |

Model adjusted for age, sex, years of education, *APOE* ε4 carrier status, hypertension, diabetes, dyslipidemia, smoking status, body mass index (BMI), and cardiovascular disease in linear mixed-effects. P values are FDR-adjusted. ADNI, Alzheimer’s disease Neuroimaging Initiative; *APOE ε4,* apolipoprotein E4; Aβ_42_, amyloid β peptide 42; P-tau, phosphorylated tau; T-tau, total tau; PDGFR, platelet-derived growth factor β; VCAM-1, vascular cell adhesion molecule-1; MMP-2, Matrix metalloproteinase 2; MMP-10, Matrix metalloproteinase 10; MMP-3, Matrix metalloproteinase 3; MMP-9/TIMP-1, Matrix metalloproteinase 9/ tissue inhibitors of metalloproteinase-1.

## Table S3 Associations of CSF biomarkers related to endothelial dysfunction with longitudinal changes in cognition

|  | MMSE | | | ADNI_MEM | | | ADNI_EF | | | ADNI_LAN | | | ADNI_VS | | |
| --- | --- | --- | --- | --- | --- | --- | --- | --- | --- | --- | --- | --- | --- | --- | --- |
|  | β | 95%CI | p value | β | 95%CI | p value | β | 95%CI | p value | β | 95%CI | p value | β | 95%CI | p value |
| PDGFRβ | -0.060 | (-0.194, 0.074) | 0.758 | -0.018 | (-0.0481, 0.0125) | 0.676 | 0.001 | (-0.027, 0.029) | 0.990 | -0.012 | (-0.041, 0.017) | 0.781 | -0.024 | (-0.061, 0.013) | 0.660 |
| Osteopontin | -0.087 | (-0.225, 0.051) | 0.660 | -0.028 | (-0.0588, 0.0034) | 0.398 | -0.019 | (-0.048, 0.009) | 0.660 | -0.011 | (-0.040, 0.019) | 0.827 | 0.024 | (-0.013, 0.061) | 0.660 |
| VCAM-1 | 0.066 | (-0.069, 0.200) | 0.741 | 0.028 | (-0.002, 0.058) | 0.382 | 0.032 | (0.005, 0.059) | 0.188 | 0.018 | (-0.010, 0.047) | 0.660 | 0.037 | (0.002, 0.072) | 0.262 |
| E-selectin | 0.024 | (-0.112, 0.159) | 0.931 | 0.032 | (0.002, 0.062) | 0.252 | 0.006 | (-0.022, 0.034) | 0.927 | 0.013 | (-0.015, 0.042) | 0.757 | 0.011 | (-0.025, 0.047) | 0.852 |
| MMP-2 | 0.099 | (-0.038, 0.236) | 0.656 | -0.014 | (-0.044, 0.017) | 0.758 | 0.006 | (-0.023, 0.034) | 0.927 | 0.016 | (-0.014, 0.045) | 0.700 | 0.006 | (-0.031, 0.042) | 0.940 |
| MMP-10 | -0.363 | (-0.488, -0.239) | 0.000 | -0.073 | (-0.102, -0.044) | 0.000 | -0.045 | (-0.072, -0.017) | 0.022 | -0.059 | (-0.086, -0.031) | 0.001 | -0.004 | (-0.040, 0.031) | 0.940 |
| MMP-3 | -0.115 | (-0.255, 0.025) | 0.500 | -0.029 | (-0.060, 0.003) | 0.386 | -0.026 | (-0.055, 0.003) | 0.386 | -0.014 | (-0.044, 0.016) | 0.741 | 0.003 | (-0.035, 0.040) | 0.966 |
| MMP-9/TIMP-1 | 0.003 | (-0.130, 0.136) | 0.990 | -0.005 | (-0.035, 0.025) | 0.940 | 0.022 | (-0.005, 0.049) | 0.503 | 0.016 | (-0.011, 0.045) | 0.676 | 0.016 | (-0.019, 0.052) | 0.758 |
| MMP-12 | -0.066 | (-0.202, 0.071) | 0.741 | 0.000 | (-0.031, 0.031) | 0.990 | -0.004 | (-0.032, 0.025) | 0.940 | 0.008 | (-0.022, 0.037) | 0.898 | 0.014 | (-0.023, 0.051) | 0.807 |
| Periostin | -0.012 | (-0.154, 0.131) | 0.965 | 0.010 | (-0.022, 0.042) | 0.852 | 0.012 | (-0.019, 0.042) | 0.805 | 0.006 | (-0.025, 0.038) | 0.927 | 0.017 | (-0.023, 0.056) | 0.769 |

Model adjusted for age, sex, years of education, *APOE* ε4 carrier status, hypertension, diabetes, dyslipidemia, smoking status, body mass index (BMI), and cardiovascular disease in linear mixed-effects. P values are FDR-adjusted. ADNI, Alzheimer’s disease Neuroimaging Initiative; *APOE ε4,* apolipoprotein E4; PDGFR, platelet-derived growth factor β; VCAM-1, vascular cell adhesion molecule-1; MMP-2, Matrix metalloproteinase 2; MMP-10, Matrix metalloproteinase 10; MMP-3, Matrix metalloproteinase 3; MMP-9/TIMP-1, Matrix metalloproteinase 9/ tissue inhibitors of metalloproteinase-1; EF, executive function; LAN, language; MEM, memory function; MMSE, Mini-Mental State Examination.

## Table S4 Associations of CSF biomarkers related to endothelial dysfunction with longitudinal changes in brain volumes

|  | Ventricles | | | Hippocampus | | | Whole brain | | | Entorhinal | | | Fusiform | | | Middle temporal | | |
| --- | --- | --- | --- | --- | --- | --- | --- | --- | --- | --- | --- | --- | --- | --- | --- | --- | --- | --- |
|  | β | 95%CI | p value | β | 95%CI | p value | β | 95%CI | p value | β | 95%CI | p value | β | 95%CI | p value | β | 95%CI | p value |
| PDGFRβ | 0.001 | (-0.008, 0.011) | 0.940 | -0.013 | (-0.032, 0.006) | 0.660 | -0.006 | (-0.016, 0.004) | 0.676 | -0.029 | (-0.054, -0.004) | 0.199 | -0.017 | (-0.036, 0.002) | 0.386 | -0.012 | (-0.031, 0.007) | 0.660 |
| Osteopontin | 0.012 | (0.002, 0.022) | 0.175 | -0.027 | (-0.047, -0.008) | 0.073 | -0.018 | (-0.028, -0.008) | 0.007 | -0.013 | (-0.039, 0.014) | 0.741 | -0.029 | (-0.048, -0.010) | 0.046 | -0.038 | (-0.057, -0.019) | 0.002 |
| VCAM-1 | -0.009 | (-0.018, 6e-04) | 0.382 | -0.002 | (-0.022, 0.017) | 0.940 | 0.011 | (0.001, 0.021) | 0.197 | 0.008 | (-0.017, 0.033) | 0.852 | 0.006 | (-0.013, 0.025) | 0.852 | 0.012 | (-0.006, 0.031) | 0.660 |
| E-selectin | -0.005 | (-0.015, 0.005) | 0.741 | 0.007 | (-0.012, 0.027) | 0.807 | 0.009 | (-8e-04, 0.019) | 0.386 | 0.001 | (-0.025, 0.026) | 0.990 | 0.010 | (-0.009, 0.029) | 0.687 | 0.012 | (-0.007, 0.031) | 0.660 |
| MMP-2 | -0.003 | (-0.013, 0.007) | 0.883 | -0.011 | (-0.031, 0.009) | 0.687 | 0.006 | (-0.005, 0.016) | 0.692 | 0.008 | (-0.019, 0.035) | 0.883 | 0.004 | (-0.016, 0.024) | 0.927 | -0.007 | (-0.028, 0.013) | 0.807 |
| MMP-10 | 0.023 | (0.013, 0.032) | 0.000 | -0.049 | (-0.067, -0.030) | 0.000 | -0.019 | (-0.029, -0.010) | 0.002 | -0.040 | (-0.066, -0.013) | 0.048 | -0.040 | (-0.059, -0.020) | 0.002 | -0.057 | (-0.075, -0.038) | 0.000 |
| MMP-3 | 0.003 | (-0.007, 0.013) | 0.852 | 0.000 | (-0.020, 0.021) | 0.990 | 0.003 | (-0.007, 0.014) | 0.852 | -0.017 | (-0.044, 0.010) | 0.660 | -0.009 | (-0.029, 0.011) | 0.758 | -0.022 | (-0.042, -0.002) | 0.210 |
| MMP-9/TIMP-1 | -0.001 | (-0.011, 0.008) | 0.943 | 0.004 | (-0.015, 0.024) | 0.924 | 0.000 | (-0.010, 0.010) | 0.990 | 0.005 | (-0.021, 0.031) | 0.930 | 0.002 | (-0.018, 0.022) | 0.957 | 0.002 | (-0.018, 0.021) | 0.963 |
| MMP-12 | -0.005 | (-0.015, 0.005) | 0.734 | -0.032 | (-0.052, -0.013) | 0.019 | -0.006 | (-0.017, 0.004) | 0.660 | -0.022 | (-0.050, 0.005) | 0.500 | -0.008 | (-0.028, 0.013) | 0.805 | 0.002 | (-0.018, 0.022) | 0.943 |
| Periostin | -0.006 | (-0.016, 0.004) | 0.676 | -0.009 | (-0.031, 0.013) | 0.777 | -0.007 | (-0.019, 0.004) | 0.660 | -0.001 | (-0.033, 0.030) | 0.990 | -0.014 | (-0.037, 0.009) | 0.676 | -0.006 | (-0.029, 0.017) | 0.910 |

Model adjusted for age, sex, years of education, *APOE* ε4 carrier status, hypertension, diabetes, dyslipidemia, smoking status, body mass index (BMI), cardiovascular disease and Intracranial volume (ICV) in linear mixed-effects. P values are FDR-adjusted.PDGFR, platelet-derived growth factor β; VCAM-1, vascular cell adhesion molecule-1; MMP-2, Matrix metalloproteinase 2; MMP-10, Matrix metalloproteinase 10; MMP-3, Matrix metalloproteinase 3; MMP-9/TIMP-1, Matrix metalloproteinase 9/ tissue inhibitors of metalloproteinase-1.

## Figure S1 Three-year time-dependent ROC curves for predicting incident AD dementia

Three-year time-dependent ROC curves for predicting incident AD dementia. Model 1 included clinical covariates, including age, sex, education, *APOE* ε4 status, hypertension, diabetes, dyslipidemia, smoking status, BMI, and cardiovascular disease. Model 2 additionally included CSF MMP-10. Model 3 included clinical covariates and AD biomarkers. Model 4 additionally included CSF MMP-10 on top of Model 3. AUC, area under the curve; ROC, receiver operating characteristic.

## Table S5 Mediating effects of CSF biomarkers related to endothelial dysfunction on the association between AD pathology and cognition in cognitively normal participants.

| **Biomarkers** | **Mediator** | **Direct effects** | | **Indirect effects** | | **Proportion of indirect effect** | |
| --- | --- | --- | --- | --- | --- | --- | --- |
|  |  | β | p value | β | p value | Proportion | p value |
| **CSF MMP-10** | **Aβ_42_** |  |  |  |  |  |  |
|  | ADNI_MEM | -0.269 | <0.001 | -0.052 | <0.001 | 0.159 | <0.001 |
|  | ADNI_EF | -0.194 | <0.001 | -0.050 | 0.002 | 0.203 | 0.002 |
|  | ADNI_LAN | -0.235 | <0.001 | -0.033 | 0.002 | 0.120 | 0.002 |
|  | ADNI_VS | -0.125 | 0.010 | -0.022 | 0.003 | 0.144 | 0.005 |
| **CSF MMP-10** | **P-tau** |  |  |  |  |  |  |
|  | ADNI_MEM | -0.209 | <0.001 | -0.112 | <0.001 | 0.349 | <0.001 |
|  | ADNI_EF | -0.164 | <0.001 | -0.079 | <0.001 | 0.324 | <0.001 |
|  | ADNI_LAN | -0.212 | <0.001 | -0.057 | 0.003 | 0.211 | 0.003 |
|  | ADNI_VS | -0.095 | 0.030 | -0.052 | 0.007 | 0.352 | 0.009 |
| **CSF MMP-10** | **T-tau** |  |  |  |  |  |  |
|  | ADNI_MEM | -0.221 | <0.001 | -0.099 | <0.001 | 0.309 | <0.001 |
|  | ADNI_EF | -0.178 | <0.001 | -0.066 | <0.001 | 0.271 | <0.001 |
|  | ADNI_LAN | -0.171 | <0.001 | -0.047 | 0.011 | 0.175 | 0.011 |
|  | ADNI_VS | 0.097 | 0.057 | -0.050 | 0.018 | 0.341 | 0.0126 |

Mediation analysis was adjusted for gage, sex, years of education, APOE ε4 carrier status, hypertension, diabetes, dyslipidemia, smoking status, body mass index (BMI), and cardiovascular disease. P values are FDR-adjusted. *APOE* ε4, apolipoprotein E4; Aβ_42_, amyloid β peptide 42; P-tau phosphorylated tau, T-tau total tau; MMP-10, Matrix metalloproteinase 10; EF, executive function; LAN, language; MEM, memory function; MMSE, Mini-Mental State Examination.
